# The Effect of Bisphenol and Its Cytotoxicity on Female Infertility and Pregnancy Outcomes: A Narrative Review

**DOI:** 10.3390/jcm13247568

**Published:** 2024-12-12

**Authors:** Eirini Drakaki, Sofoklis Stavros, Dimitra Dedousi, Anastasios Potiris, Despoina Mavrogianni, Athanasios Zikopoulos, Efthalia Moustakli, Charikleia Skentou, Nikolaos Thomakos, Alexandros Rodolakis, Peter Drakakis, Ekaterini Domali

**Affiliations:** 1 First Department of Obstetrics and Gynecology, Alexandra Hospital, Medical School, National and Kapodistrian University of Athens, 115 28 Athens, Greece; eirinidrak@med.uoa.gr (E.D.); dimitradedousi@hotmail.com (D.D.); dmavrogianni@med.uoa.gr (D.M.); nthomakos@med.uoa.gr (N.T.); arodolak@med.uoa.gr (A.R.); kdomali@yahoo.fr (E.D.); 2 Third Department of Obstetrics and Gynecology, University General Hospital “ATTIKON”, Medical School, National and Kapodistrian University of Athens, 124 62 Athens, Greece; sfstavrou@med.uoa.gr (S.S.); thanzik92@gmail.com (A.Z.); pdrakakis@med.uoa.gr (P.D.); 3 Laboratory of Medical Genetics, Faculty of Medicine, School of Health Sciences, University of Ioannina, 451 10 Ioannina, Greece; thaleia.moustakli@gmail.com; 4 Department of Obstetrics and Gynecology, Medical School, University of Ioannina, 451 10 Ioannina, Greece; haraskentou@uoi.gr

**Keywords:** bisphenols, bisphenol A (BPA), endocrine disrupting compounds (EDCs), female infertility, perinatal outcomes

## Abstract

Bisphenols, particularly bisphenol A (BPA), are among the most thoroughly investigated endocrine disrupting chemicals (EDCs). BPA was the first synthetic estrogen to be identified, exerting its estrogenic effects through interaction with human estrogen receptors (ERs). The aim of the present narrative review is to summarize the most recent literature regarding the adverse effects of bisphenols on female fertility and pregnancy outcomes. A review of the literature in the PubMed/Medline and Scopus databases was conducted in November 2024 and 15 studies were included in the present review. BPA levels were higher in women with diminished ovarian reserve, polycystic ovary syndrome, and recurrent miscarriages. Furthermore, one study showed a significant association between BPA levels and the onset of gestational diabetes mellitus. Higher levels of BPA are associated with disruptions to the female reproductive system, such as ovarian function, reduced number of antral follicles, and lower anti-Müllerian hormone (AMH) levels. Bisphenols A and S were associated with an increased risk of developing gestational diabetes mellitus. Bisphenols A and F were correlated with an increased risk of lower birth weight and bisphenol F seemed to be associated with an increased risk of preterm delivery. Ultimately, further research is necessary to fully understand the extent of the harmful effects that bisphenols have separately and as mixtures on the female reproductive system.

## 1. Introduction

Infertility constitutes a major medical condition with profound personal and societal implications [1]. According to the latest definition by the American Society of Reproductive Medicine, infertility is a disease, condition, or status of the reproductive system characterized by the failure to achieve a pregnancy after regular and unprotected sexual intercourse or the need for medical intervention [2]. Globally, infertility is estimated to affect approximately 8 to 12% of couples of reproductive age [3]. Τhe rate of fertility reduction has accelerated significantly over the past few decades [1]. Unfortunately, fertility is expected to continue to decrease in the near future.

Infertility is classified into primary and secondary infertility. Primary infertility refers to couples who have never succeeded in conceiving, whereas secondary infertility describes difficulties in conceiving after having previously achieved pregnancy, whether resulting in a live birth or miscarriage [4]. Secondary infertility is the most frequent type of female infertility and it is more prevalent in areas with high rates of unsafe abortions and inadequate maternal healthcare [3]. In developed countries, approximately one in seven women of reproductive age experience infertility, while, in developing countries, the rate is about one in four couples. Roughly half of all cases of infertility are attributed to female reproductive issues [5]. Female infertility could result from the advanced age of women at the time of conception, as well as from various medical conditions including premature ovarian failure, polycystic ovary syndrome (PCOS), endometriosis, uterine fibroids, or endometrial polyps [6,7]. Additional conditions that have the potential to impact female fertility include reproductive tract infections, lifestyle related factors, and various exposures to environmental chemicals [3]. Over the past few decades, there has been increasing interest in the impact of endocrine disruptors on the female reproductive system and pregnancy [8].

Endocrine disrupting chemicals (EDCs) are exogenous chemical substances that can interfere with normal endocrine functions. EDCs can disrupt hormonal balance by mimicking natural hormones such as estrogens and/or androgens, blocking their actions, or altering the concentrations of endogenous hormones within the body. In particular, EDCs can trigger tissue-specific estrogenic effects by acting as either agonists or antagonists of estrogen receptors (ERs), disrupting ERα-dependent transcriptional signaling pathways [9]. They may also interfere with ERα-mediated transcription by influencing interactions between ERs and other nuclear receptors, as well as through modulation of growth factors [9]. This disruption can lead to a variety of health issues such as reproductive and developmental disorders [8,10]. Thorough research has shown that EDCs can negatively impact distinct characteristics of female fertility, including the number of follicles, ovulation, meiosis, and uterine implantation of the embryo [3]. High levels of endocrine disruptors could be found in various sources including industrial chemicals, pesticides, plastic bottles, food packages, cosmetics, and even in some natural products [11]. Exposure to these chemicals can occur through ingestion of contaminated food, inhalation, or skin contact. Among the major endocrine disruptors are bisphenols, which have the potential to affect the progression of pregnancy and the development of the fetus [8,12].

Bisphenols (BPs), and especially bisphenol A (BPA), are widely used in the production of polycarbonate plastics and epoxy resins. Therefore, BPs are present as ingredients in various everyday products including food packaging, baby plastic bottles, dental materials, and the inner lining of food cans [13]. Consequently, the main route of exposure to BPs is through dietary intake [14]. BPA (2,2-bis (4-hydroxyphenyl) propane) is synthesized through a condensation reaction between phenol and acetone in the presence of an ion-exchange catalyst. BPA was the first synthetic estrogen to be identified, exerting its estrogenic effects through interaction with human estrogen receptors (ERs), particularly estrogen receptor alpha (ERα) [14]. BPA activates signaling pathways associated with estrogen receptors ER-α (alpha) and ER-β (beta), mimicking the activity of natural estrogen [9]. These estrogenic effects could cause cellular changes in estrogen-sensitive tissues, potentially leading to disruptions in normal hormonal functions and health issues, especially within the reproductive and endocrine systems. BPA can also cross the placenta and amniotic fluid, leading to its accumulation in fetal tissues. Exposure to this endocrine disruptor can potentially affect the development and health of the fetus [15,16]. It is worth noting that several studies have demonstrated that exposure to high levels of BPA, which can be detected in both serum and urine, is associated with disruptions to the female reproductive system, such as disruption of ovarian function, irregular menstrual cycles, reduced number of antral follicles, and lower anti-Müllerian hormone (AMH) levels. These factors could potentially contribute to infertility [13].

Due to its cytotoxicity and reproductive toxicity, BPA has been removed from many products and substituted by alternatives such as BPS (4,40-sulfonyldiphenol) and BPF (4,40-dihydroxydiphenylmethane), which are considered to be less detrimental to the human body. BPS is produced by combining phenol with sulfur trioxide, while in the synthesis of BPF, formaldehyde acts as the solvent and a Brønsted acidic ionic liquid serves as the catalyst [17,18]. However, because of their structural resemblance to BPA, these analogues have a negative impact on the reproductive system by exerting estrogenic and antiandrogenic effects [13]. The present review aims to summarize the most recent literature regarding the negative effects of BPs on female fertility and pregnancy outcomes. The studies included in this review provide additional information regarding the most recent data that associate exposure to BPs with the occurrence of female infertility and adverse pregnancy outcomes.

## 2. Literature Research

A thorough literature investigation was performed in the Medline/Pubmed and Scopus databases in November 2024 with the aim of identifying relevant studies and articles regarding the negative impact of bisphenols on female fertility and pregnancy outcomes. The search query used included keywords like “Bisphenol*”, “Bisphenol A”, “BPA”, “Bisphenol S”, “Bisphenol F”, “Endocrine Disrupting Chemicals”, “EDCs”, “Female infertility”, “ovarian reserve”, “polycystic ovary syndrome”, “PCOS”, “Pregnancy outcomes”, “preterm birth”, “birth weight”, and “gestational diabetes mellitus”. These search terms were either used as presented, separately, or in combination with the help of the Boolean administrators (OR, AND). Neither time limit nor other filters were applied in either database. The search was further refined by including studies that directly investigated the impact of bisphenol exposure. Additionally, the “snowball literature searching method” was applied to identify further relevant sources from the reference lists of selected articles. A formal risk of bias and quality assessment was not performed due to narrative nature of this review.

The inclusion criteria for the present review included studies that met the following criteria: (1) examined the cytotoxicity of bisphenols and their effects on female fertility and pregnancy outcomes; (2) were patient-control studies, cohort studies, and cross-sectional studies with adequate primary data (number of subjects per population and statistical for comparison *p*-value or q-value and/or foldchange). Similarly, the exclusion criteria included studies that (1) did not relate to bisphenols or their effects on female reproductive system, (2) did not provide adequate data (number of subjects per population and statistical for comparison *p*-value or q-value and/or foldchange) for extraction, and (3) were written in a language other than English.

## 3. Results

The present review includes fifteen studies. Regarding the effect of bisphenols in female infertility, two cross-sectional and two cohort studies assess the impact of BPA exposure on ovarian reserve, two case-control studies demonstrate the association between bisphenols and the occurrence of PCOS, and there is one cross-sectional study regarding the possible correlation between exposure to BPA and BPS and the occurrence of oxidative stress along with homeostatic imbalance. Regarding the impact of bisphenols on pregnancy outcomes, there are two case-control studies and one cohort study that examine the association between exposure to BPA and BPS during pregnancy and the occurrence of GDM; four cohort studies exploring the impact of bisphenols A, S, and F on birth weight; and one cohort study demonstrating the impact of BPF exposure on the risk of preterm delivery. These studies were published between 2020 and 2024. Table 1 summarizes the key clinical outcomes of each study included in this review.

Τhe study conducted by Park et al., published in 2021 [19], aimed to assess the potential correlation between exposure to bisphenol A (BPA) among women and diminished ovarian reserve (DOR). In this cross-sectional study, 307 Korean women of reproductive age were included. Participants were classified into two groups, determined by anti-Müllerian hormone (AMH) percentiles corresponding to age-specific serum AMH concentrations. Τhe DOR group consisted of 93 women with AMH levels lower than 25%, while the non-DOR group included 214 women who had serum AMH levels greater than 25%. Individuals had an average age of 36.8 ± 4.4 years, and their mean BMI was 22.4 ± 3.1 kg/m^2^. The researchers further evaluated the mean BPA levels in both groups. In the DOR group, the mean concentration of urinary BPA ranged from 1.89 ± 2.17 μg/g, while, in the non-DOR group, the average BPA concentration was 1.58 ± 1.08 μg/g. Therefore, the average BPA concentration was observed to be significantly elevated in the group that included women with diminished ovarian reserve. Furthermore, the researchers detected that BPA levels were elevated while serum AMH concentrations adjusted for BMI decreased mainly in women aged 40 to 44 years. The results of the logistic regression analysis suggested a higher incidence of infertility among women with BPA levels at or above the 90th percentile, with the odds ratio (4.248) being statistically significant after accounting for factors such as age, first menstruation age, oral contraceptives, number of pregnancies, and waist measurement. Finally, no association was observed between participants’ exposure to BPA and the occurrence of fibroids, endometriomas in the ovaries, or adenomyosis [19].

Another cross-sectional study, published in 2021 by Czubacka et al. [20], aimed to determine the effects of environmental exposure to BPA on ovarian reserve. A total of 511 women who visited a fertility clinic due to difficulties in conceiving were included in the study. Women with long-term conditions that could affect ovarian function such Fragile X syndrome or chromosomal abnormalities were excluded from the research. The main cause of infertility was attributed to male factors, with idiopathic infertility and female factors following. The mean age of the participants was 33.30 ± 3.69 years, while their average BMI was 23.18 ± 3.80 kg/m^2^. Blood and urine samples were collected from all subjects. The researchers evaluated the ovarian reserve by determining the antral follicle count (AFC) as well as the levels of estradiol (E2), follicle-stimulating hormone (FSH), and anti-Müllerian hormone (AMH) between the second and fourth day of the menstrual cycle. BPA levels in urine were determined using gas chromatography ion-trap mass spectrometry. The study results revealed that BPA was present in 97% of the participants’ urine samples with BPA levels recorded at 1.38 ± 2.34 ng/mL and SG-adjusted BPA levels at 1.60 ± 2.15 ng/mL. The levels of bisphenol A in urine showed a negative correlation with the levels of AMH (*p* = 0.02) and AFC (*p* = 0.03). However, the BPA urinary concentration did not correlate with other evaluated ovarian reserve factors such as FSH and E2 [20].

Similar results regarding the effect of bisphenols A, F, and S on ovarian reserve were presented in a cohort study from Shenyang, China. The authors showed that higher BPA (OR = 7.112, 95% CI: 1.247–40.588, *p* = 0.027) and BPS (OR = 6.851, 95% CI: 1.241, 37.818, *p* = 0.027) concentrations were significantly associated with a seven-fold higher risk of diminished ovarian reserve. Furthermore, the authors showed that there was a significant negative association between BPS levels and AMH (*p* = 0.010) [21]. The same results also applied also to the antral follicle count. The authors in another publication from the same study sample demonstrated that higher urinary concentrations of BPA, BPF, and BPS were significantly associated with lower AFC (β = 0.016; 95% CI: 0.025, 0.006 in BPA; β = 0.017; 95% CI: 0.029, 0.004 in BPF; β = 0.128; 95% CI: 0.197, 0.060 in BPS). Furthermore, a quantile increase in the bisphenols mixture was negatively associated with AFC [22].

In a 2023 study, the scientific team of Zhan et al. [23], aimed to determine the effects of BPs on female fertility focusing on their association with the occurrence of polycystic ovary syndrome (PCOS). A multi-center hospital-based case-control study was performed, including a total of 733 Chinese women. The participants were classified into two groups. The first group was defined as cases, consisting of 321 women who had been diagnosed with infertility due to PCOS according to the Rotterdam diagnostic criteria. The age of the cases ranged from 20 to 40 years (mean age 29 years). The control group included 412 women without reproductive disorders who had visited the same fertility clinics because of male infertility and underwent donor artificial insemination (AID). The mean body mass index (BMI) of the participants was 23.06 kg/m^2^, while approximately 33% of these women were overweight or obese. The BMI of the cases was significantly higher compared to the control group (*p* < 0.01). Using liquid–liquid extraction paired with high-performance liquid chromatography–tandem mass spectrometry (HPLC-MS/MS), the concentrations of seven bisphenol variants were measured in urine samples. The examined bisphenol analogs were BPA, BPAF, BPAP, BPB, BPP, BPS, and BPZ. Additionally, the researchers analyzed the levels of FSH, LH, prolactin, estradiol, and testosterone in the serum from venous blood samples taken during the first three days of the menstrual cycle. The age of the women, their body mass index (BMI), alcohol intake, smoking, educational and occupational status, and the location of the study were identified as potential confounding factors in the relationship between the occurrence of polycystic ovary syndrome and exposure to bisphenols. The research team used odds ratios with a 95% confidence interval to demonstrate the association between bisphenol levels and the increased risk of developing PCOS. The analysis revealed that the urine samples of the cases exhibited statistically significant higher levels of the seven bisphenol analogs compared to those of the control group. Bisphenol A (BPA) presented the highest concentration with a measurement of 1.14 μg/g Cr. The researchers observed that there was a statistically significant relationship between the presence of the bisphenols BPA (aOR 1.09; 1.05–1.14), BPAF (aOR = 1.07; 1.02–1.13), BPS (aOR = 1.18; 1.10–1.25), and BPZ (aOR = 1.15; 1.08–1.22) in urine samples and the risk of developing polycystic ovary syndrome (PCOS). Quantile-based g computation (QGC) analysis was performed, revealing that exposure to a combination of seven bisphenol analogs was significantly linked to an increased risk of PCOS (aOR = 1.26; 1.12–1.45), largely attributed to BPS, BPZ, and BPAF. Hormonal analysis revealed that women with PCOS had significantly increased levels of LH and testosterone, but significantly decreased FSH and estradiol levels compared to the control group (*p* < 0.01). Furthermore, a significant positive correlation between BPA, BPF, BPS, and testosterone levels was demonstrated. Finally, women classified as overweight or obese exhibited a stronger link between bisphenol variants and PCOS compared to those who maintained a normal body weight. There was no significant relationship between the concentrations of bisphenol variants and the use of plastic household items [23].

A similar negative association between BPA and PCOS was presented in the case-control study by Patel et al. The authors demonstrated that the BPA levels in the peripheral blood sampling of the PCOS group were significantly higher than the controls (102.15 ± 0.1 ng/mL vs. 61.35 ± 50.13 ng/mL, *p* < 0.0001). Furthermore, a significant positive correlation between bisphenol A and luteinizing hormone (LH) levels (r = 0.23, *p* = 0.03) was observed [24].

The study by Liang et al., published in 2020 [25], sought to investigate the possible correlation between exposure to BPA and BPS and the occurrence of oxidative stress along with homeostatic imbalance, which represent factors linked to the occurrence of recurrent spontaneous miscarriage. A total of 111 women with a history of recurrent spontaneous abortion participated in this cross-sectional study. Women who experienced recurrent miscarriages due to other causes, such as active infections, chromosomal or anatomical anomalies, endocrine disorders, and autoimmune diseases, were excluded from the study. The mean age of the women who were included in the study was 28 years (range 26 to 31 years) with a BMI of 22.0 kg/m^2^ (range 20.0 to 24.7 kg/m^2^). Among them, 88.3% (98/111) had a regular menstrual cycle, while 25.2% (28/111) had previously given birth. The concentrations of BPA and BPS were determined in urine samples using ultra-performance liquid chromatography with tandem mass spectrometry (UPLC-MS/MS). The detection limits (LOD) were 0.1 ng/mL for BPA and 0.01 ng/mL for BPS, respectively. Furthermore, the concentrations of two major biomarkers of oxidative stress, 8-hydroxy-deoxyguanosine (8-OHdG) and 8-isoprostane, were identified in the specific urine samples. The serum levels of a range of cytokines which serve as biomarkers of immune homeostasis were also evaluated through ECL-ELISA. BPA was present in 99.1% of the urinary samples collected from the 111 women. On average, the concentrations of urinary BPA and creatinine-adjusted BPA were 0.95 ng/mL and 1.41 mg/g creatinine, respectively. 8-hydroxy-deoxyguanosine (8-OHdG) was detected in 100% of the urinary samples, while 8-isoprostane was present in 98.2%, with median concentrations of 0.02 mg/g creatinine for 8-OHdG and 1.49 mg/g creatine for 8-isoprostane. Levels of cytokines, both inflammatory and anti-inflammatory, were detectable in between 9.0% and 100% of the serum samples from all studied women. To evaluate the association between bisphenol exposure levels and biomarkers of oxidative stress and immune balance, multivariable linear regression models were utilized. BPA demonstrated a significant correlation with elevated concentrations of both 8-isoprostane and IFN-γ, while no statistical relationship was noted between BPA and 8-OHdG. Additionally, no significant association was observed between BPS concentrations and oxidative stress or immunology [25].

The cohort study by Soomro et al., published in 2024 [26], examined the association between exposure to bisphenol A and other endocrine disrupting chemicals (EDCs) in pregnant women during the second trimester and the occurrence of gestational diabetes mellitus (GDM). Among the 420 women involved in the research, 15 (3.57%) were diagnosed with diabetes during pregnancy. Regarding the participants’ ages, 213 women (50.71%) were in the 30 to 34 age range and 113 (26.90%) were over the age of 35. A total of 223 women reported that they were experiencing their first pregnancy. Almost all women refrained from smoking during their pregnancy, while only 4.52% reported alcohol consumption. Urine samples were collected to determine the concentrations of bisphenol A and S. The participants’ blood sugar levels were evaluated between the 24th and 28th weeks using the recommended screening for GDM. Childbearing history, maternal age, BMI, and the educational background of the pregnant women were considered as potential adjustment variables. The results of the study showed that bisphenol A (BPA) had a detection ratio of 92.3%, whereas bisphenol S (BPS) had a detection ratio of 58.6%. Moreover, the minimum detectable concentrations of BPA and BPS were found to be 0.32 μg/L and 0.10 μg/L, respectively. In models examining single exposures, multiple logistic regression analysis indicated a significant link between bisphenol A (BPA) and a greater risk of developing gestational diabetes mellitus (GDM) in both unadjusted (OR: 1.69, 95% CI: 1.03–2.78) and adjusted analyses (AOR: 1.70, 95% CI: 1.00–2.91). On the other hand, no association was found between bisphenol S and the occurrence of diabetes during the second trimester of pregnancy [26].

On the contrary, a cohort study from China examined the effect of bisphenol exposure during pregnancy and the development of GDM. In this study, 500 women were included (100 women with GDM and 400 matched controls) and bisphenol exposure was measured via blood sampling in the first trimester. The study results showed a statistically significant positive correlation between BPS exposure and the development of GDM, especially in medium- and high-exposure cases (for medium exposure: OR: 1.71, 95% CI: 0.99–2.96 and aOR: 1.77, 95% CI: 1.01–3.13; for high exposure: OR: 1.68, 95% CI: 0.96–2.94 and aOR: 1.68, 95% CI: 0.95–2.99). It should be noted that, in this study, BPA exposure was negatively correlated with GDM [27].

The negative effects of BPS exposure regarding the development of GDM were reported by another case-control study with a population from the United States. In this study, urine samples taken in the first and second trimester from 333 pregnant women (111 with GDM and 222 matched controls) were assessed. Study results showed that GDM cases had higher detection frequency of BPA in the second trimester (79.6 vs. 66.7%) and higher cumulative levels of BPS across the two trimesters (145.7 vs. 103.9 ng/mL × day) compared to the control group. Regarding the odds of developing GDM, the authors showed that first-trimester urinary BPS was associated with higher risk (aOR: 2.12, 95% CI 1.00–4.50) [28].

Regarding the effect of bisphenols on birth weight, a recent cohort study from the NIH Environmental influences on Child Health Outcomes (ECHO) Program included 3619 singleton pregnancies with at least one bisphenol measurement during pregnancy. The authors showed that bisphenol S exposure in the third trimester was associated with an increased risk of delivering a small for gestational age newborn (OR 1.52, 95% CI 1.08, 2.13). Furthermore, the study results revealed a significant association between bisphenol F exposure in first and third trimester and Low Birth Weight (less than 2500 g). Interestingly, this study showed no positive association between BPA exposure and birth weight, mainly due to the increased usage of replacement bisphenols F and S [29]. In a cohort study from China, both BPA and BPF exposure, measured in the third trimester, were significantly correlated with lower birth weight. BPS exposure showed no significant correlation [30].

Hong et al. [31], in their cohort study, showed that BPA levels in the fetal cord blood had a significant linear association with the head circumference to abdominal circumference ratio. Furthermore, in the same study the authors showed that maternal and fetal BPA levels were significantly higher in the fetal growth restricted group than in the normal birthweight group [31]. Lastly, Sol et al. [32], in a population-based cohort which was embedded in the Generation R Study, demonstrated that bisphenol S was associated with larger fetal head circumference and fetal weight. Furthermore, bisphenol S and F exposure in the first trimester were also associated with a lower risk of being born small for gestational age [32].

Finally, regarding the effect of BPF on preterm birth, Liang et al., in their study, showed that bisphenol F (BPF) was positively correlated with an increased risk of preterm birth (OR = 1.73, 95% CI: 1.18–2.55). Moreover, the authors showed that BPA concentrations were also associated with preterm birth, especially in the subgroup of female infants (OR = 1.30, 95% CI: 1.02–1.64) [33].

## 4. Discussion

In modern societies, the rapid development of industry has led to the daily exposure of individuals to several toxic chemical compounds that may adversely affect the endocrine system. Among these substances, bisphenols, and especially bisphenol A, are frequently used in plastic production and can be found in high concentrations in numerous consumer products, making them some of the most well-studied endocrine disrupting chemicals (EDCs). Lately, there has been increasing concern regarding the negative impact of bisphenols on female fertility. In the present review, we aim to summarize the most recent data regarding the effects of bisphenols on the female reproductive system and pregnancy outcomes.

The study conducted by Park et al. [19], revealed that the exposure of reproductive-age Korean women to BPA could have detrimental effects on ovarian reserve, leading to infertility. The scientific team demonstrated that the mean urinary BPA concentration was significantly elevated in the group that included women with diminished ovarian reserve (DOR group). These findings are in agreement with those of Zhou et al., which demonstrated that higher BPA levels in urine correlated with a decrease in antral follicle count (AFC) [34]. However, their findings were not statistically significant. It is important to note that bisphenols, particularly bisphenol A, exert a toxic effect on the ovaries by interfering with various pathways, such as those of oxidative stress, apoptosis, and folliculogenesis [35]. Furthermore, BPA has been associated with alterations in the structure of the fallopian tubes and uterus, along with disruptions in the expression of GnRH and kisspeptin which play a crucial role in the reproductive hormonal system [35,36]. The results of this research could be limited by the small sample size and the fact that bisphenol A has a brief half-life, while exposure to different endocrine disruptors occurs over a long period. However, it is worth mentioning that the study by Park et al. consisted of women who participated voluntarily and did not comprise patients from infertility clinics. Therefore, they could be seen as more representative of the general population.

The findings of the above study are also supported by Czubacka et al. [20], who presented the adverse effects of bisphenol A on ovarian reserve. The researchers evaluated the relationship between BPA exposure and four indicators of ovarian reserve, including AMH, AFC, E2, and FSH. A negative correlation was observed between AMH, AFC, and BPA levels, while no association was found between E2 and FSH with BPA concentrations. It is worth mentioning that anti-Müllerian hormone (AMH) and antral follicle count (AFC) are considered the most specific and reliable indicators of ovarian sufficiency [37]. The findings of Czubacka et al. [20] are consistent with the results of previous studies that attempted to determine the effects of BPA exposure on the ovarian reserve of reproductive-age women. Specifically, Souter et al., through a prospective cohort study that included women undergoing infertility treatment, observed a significant correlation between high BPA levels and a reduction in AFC [38]. Moreover, Cao et al. reached a similar deduction in their study demonstrating that, in the follicular fluid of women with diminished ovarian reserve, BPA concentrations were significantly increased, while the levels of AMH and E2 were decreased compared to healthy women [39]. Although the study by Czubacka et al. [20] involves a considerable number of participants, its findings might be limited and not generalizable in the broad population since it focuses on women experiencing infertility problems from a particular clinic. Finally, the study does not describe the mechanisms by which bisphenol A exerts its harmful effects on the ovarian reserve parameters.

The research conducted by Zhan et al. [23] indicates that exposure to bisphenol A (BPA) and its analogs, such as BPAF, BPAP, BPP, BPS, and BPZ, is correlated with a significantly higher risk of developing polycystic ovary syndrome (PCOS), especially among women who are overweight or obese. However, it should be mentioned that the BMI in the case group was significantly higher than the controls. These results are supported by the studies of Konieczna et al. and Jurewicz et al., which concluded that bisphenol A and bisphenol S levels are associated with increased odds of developing polycystic ovary syndrome, respectively [40,41]. Given its estrogenic activity, BPA may interact with various endocrine systems and metabolic pathways, while bisphenol analogs may exert similar effects on metabolic pathways, promoting insulin resistance and hyperinsulinemia through chronic inflammation, which in turn leads to increased secretion of GnRH and LH, as seen in PCOS [42]. Polycystic ovary syndrome (PCOS) is the most common endocrine disorder of women of reproductive age and its prevalence ranges between 5% and 21% globally [23]. According to the Rotterdam criteria, polycystic ovary syndrome (PCOS) could be diagnosed when at least two of the following three criteria are present: (1) clinical or biochemical hyperandrogenism, (2) irregular or absent ovulation, (3) presence of polycystic ovarian morphology observed via ultrasound imaging [43]. The researchers also noted a significant positive correlation between BPA, BPF, BPS, and testosterone levels. These findings are consistent with the results of the study conducted by Kandaraki et al., which demonstrated that women with PCOS exhibited significantly elevated BPA serum levels, which were also positively correlated with serum levels of testosterone and androstenedione [44]. The findings of Jurewicz et al. further support the association between higher bisphenol A concentrations and increased serum testosterone levels [40]. In vitro studies have shown that BPA and its analogs induce the production of androgens by the ovaries through disruptions to the activity of 17α-hydroxylase, leading to hyperandrogenism, which is a typical trait of PCOS [45,46].

According to Liang et al., exposure to bisphenols A and S may be associated with oxidative stress and immune system imbalance in women with unexplained recurrent spontaneous abortion (URSA). Based on the latest definition provided by the American Society for Reproductive Medicine and the European Society of Human Reproduction and Embryology, recurrent pregnancy loss is defined as the loss of two or more consecutive pregnancies before the 24th week of pregnancy [47,48]. Among the factors that have been implicated in the incidence of recurrent miscarriage, recent studies have focused on the potential impact of various chemical substances on the occurrence of this major reproductive health issue. The researchers observed that BPA levels were associated with increased levels of a biomarker of oxidative stress, 8-isoprostane. In the case of 8OHdG, no similar positive correlation was found. At the same time, the research team concluded that the increased concentrations of BPA were also related to the elevated levels of the pro-inflammatory cytokine IFN-γ. The study showed that BPA concentration in the urine samples of women experiencing unexplained recurrent abortion was 1.41 mg/g creatinine, which was higher than those in the control group. These results are consistent with the studies by Shen et al. and Peng et al., which support the higher levels of bisphenol A in women with URSA [49,50]. Although the mechanism by which exposure to bisphenols may lead to miscarriages has not been clarified yet, scientific evidence suggests that oxidative stress and immune imbalance are factors that could lead to various disorders in the female reproductive system. Specifically, BPA can activate estrogen receptors, leading to the production of reactive oxygen species (ROS) and several inflammatory cytokines, including IFN-γ [51]. The subsequent inflammatory responses mediated by these cytokines interfere with the normal development of the embryo and ultimately result in miscarriage [52]. According to the study, BPS levels in women with URSA were measured at 0.07 mg/g creatinine. Despite the lack of adequate evidence linking BPS exposure to an increased risk of miscarriage, further research is necessary to explore the potential role of BPS in miscarriage occurrence. The study showed that BPS, which is the main substitute for BPA, demonstrates a positive correlation with IL-10. IL-10 plays an important role in maintaining a normal pregnancy by suppressing the production of pro-inflammatory cytokines. However, elevated levels of IL-10 are associated with placental development disorders and consequently adverse pregnancy outcomes [53,54].

Another potential effect of bisphenols during pregnancy concerns their impact on the increased possibility of developing gestational diabetes mellitus (GDM). GDM, which is characterized by impaired glucose tolerance that occurs or is recognized for the first time during pregnancy, is one of the most frequent complications during pregnancy, with a growing prevalence observed worldwide. Gestational diabetes is linked to gynecological and neonatal complications resulting from elevated birth weight, as well as potential long-term health issues for both the mother and child, including type 2 diabetes and cardiovascular diseases [55]. Soomro et al. aimed to investigate the potential impact of bisphenols and other endocrine disrupting chemicals (EDCs) on the development of GDM. Their results revealed a significant correlation between BPA and the onset of diabetes during pregnancy [26]. These findings are consistent with the prospective study by Zhu et al., who observed a positive association between BPA levels and the development of GDM in early and mid-pregnancy [56]. In this study, a positive association was also found between BPS concentrations in the first trimester of pregnancy and the onset of diabetes [56]. In contrast to this finding, Soomro et al. [26] did not identify any association between bisphenol S and the occurrence of GDM. These differences may be attributed to variations in sample size or in the different levels of exposure to BPS. In addition, it should be noted that the limited number of women diagnosed with gestational diabetes (*n* = 15) in the present study could represent a limitation, as the researchers were unable to stratify the results by fetal gender. Finally, another limitation of the study by Soomro et al. could be the restriction of using a single urine or blood sample from each woman in the second trimester of pregnancy, which resulted in the inability to assess variability in bisphenol levels throughout the entire pregnancy, as well as to identify time points when the possibility of exposure of pregnant women to these endocrine disruptors could be greater and related to the onset of gestational diabetes. The same negative correlation between BPA exposure and GDM was reported by a case-control study with 301 pregnant women from the United States. In this case-control study, BPA was negatively associated with GDM (aOR: 0.61, 95% CI: 0.37–0.99). However, in this study the study sample was relatively small, and the two comparing groups were not matched [57].

The study conducted by Cantonwine et al., published in 2010 [58], suggested that women who delivered prematurely (<37 weeks) had higher concentrations of BPA in their urine samples compared to those who delivered after 37 weeks. However, the study has several limitations, including a small sample size, single urine collection, and the use of the participants’ last menstrual period for determining gestational age [58]. A study by Ye et al., demonstrated that women who developed preeclampsia had significantly higher BPA levels in their serum samples between the 16th and 20th weeks of pregnancy. Preeclampsia is characterized by high blood pressure during pregnancy (systolic ≥ 140 mmHg and/or diastolic ≥ 90 mmHg) along with proteinuria (>300 mg/24 h) occurring after 20 weeks of gestation. However, additional research is necessary to further explore the link between BPA exposure and the development of preeclampsia [59].

Similar negative effects of bisphenol exposure have been also shown in analysis of the placenta of fetal growth restricted fetuses. Cao et al. showed that BPA levels and estrogen levels were significantly higher (*p* < 0.001) in the placenta of FGR fetuses compared to normal weight fetuses, pointing to the cytotoxic events induced by bisphenols [60].

Studies show conflicting results about the influence of BPA on steroidogenesis-related genes; Liu et al. [61], show that, in KGN cells, low concentrations of BPA induce increased expression of *FOXL2*, a transcription factor related to ovarian function. BPA is also associated with elevated aromatase expression and E2 production, according to Kwintkiewicz et al., [62] while Watanabe et al. [63] showed that higher concentrations of BPA (50 μM) downregulated *CYP19* gene transcription. Shi et al. [64] showed that exposure to BPA may induce a decrease in ferredoxin and ferredoxin reductase expression and consequently regulate progesterone synthesis. They also showed that exposure to BPA may also lead to the inhibition of the testosterone-related genes *HSD17B2* and *HSD17B3* and the E2 production-related gene *HSD17B* while simultaneously upregulating p450 oxidoreductase expression.

Additionally to hormone receptor binding, BPA may induce molecular changes in endocrine mechanisms by altering the epigenome [65]. Dolinoy et al. suggested that maternal exposure to BPA may influence offspring by altering their epigenome [66]. It is also known that, during embryonic development, the genome undergoes extensive epigenetic reprogramming. Zhang showed that DNA methylation of the imprinted genes *Igf2r*, *Peg3*, and *H19* showed a decrease of 12–30% in fetal mouse germ cells when they were treated with high doses of BPA [67], while Iqbal et al. found no changes in DNA methylation of germ cells following BPA in utero exposure [68]. Chao et al. tested the potential effects of BPA on DNA methylation during mouse oocyte growth and reported decreased DNA methylation at *Peg3* and *Igf2r* when oocytes were exposed to BPA [69].

Another organ related to disease risk in offspring is the placenta, as it acts as a barrier to maternal xenobiotics, hormones, and pathogens. Concentrations of 1–104.4 ng/gr were measured in the placenta tissue and male fetuses accumulated significantly higher levels of BPA compared to female fetuses [70]. BPA can directly influence the placenta, resulting in abnormal development of trophoblast cells. This disruption could impact hCG secretion from early trimester trophoblasts and increase cell apoptosis [71]. Consequently, if the placental function is impaired by EDCs, fetal development may be compromised. Kang et al. showed that in utero BPA exposure reduced the expression of *Rtl1* in the placenta [72], while Susiarjo et al. demonstrated that BPA exposure significantly reduced DNA methylation [73].

Although the results of the studies included in this review are consistent with findings from previous research, they could be limited by specific factors, including variations in the methods used to detect bisphenol levels, small sample sizes, and differences in the ethnic backgrounds of the participants. Additionally, the available literature on the effects of bisphenol analogues, such as BPS and BPF, on female fertility is limited, which could raise concerns about the reliability of the findings. Consequently, the effects of bisphenols on female fertility and pregnancy outcomes requires further study.

In the future, further research is required to investigate the effects of the substitutes of bisphenol A, such as bisphenols S and F, on female reproductive health, as they share structural similarities with BPA, which has been thoroughly studied for its toxic impact on the reproductive system. Additionally, greater emphasis should be placed on the molecular mechanisms through which BPA and bisphenol analogs exert their negative effects on the female reproductive system, affecting hormone functions, ovaries, or other procedures such as embryo implantation. Finally, future studies should also focus on the changes in lifestyle and nutrition that women of reproductive age or pregnant women could make in order to reduce their exposure to endocrine disrupting chemicals such as bisphenols.

## 5. Conclusions

Overall, the current research evidence presents a variety of effects of bisphenols, particularly bisphenol A, on female fertility and pregnancy outcomes. While the impact of BPA on both female infertility and pregnancy outcomes has been more extensively studied, the negative effects of other bisphenol analogues are debatable due to the limited number of studies. In the present review, we sought to provide the most recent evidence on the effects of analogues S and F on gestational diabetes, birth weight, and preterm birth. Nevertheless, further research is necessary to fully understand the extent of the harmful effects that bisphenols have separately and as mixtures on the female reproductive system.

## Figures and Tables

**Table 1:** Studies included in this review.

Authors, Year	Study Design	Study Location	Sample	Sample Size	Mean Age	Ethnicity	Main Outcome
	Studies investigating the effect of bisphenols on female fertility						
Park et al., 2021 [19]	Cross-sectional study	Ewha Womans University Mokdong Hospital in Seoul of Korea	Urine samples	307 women	36.8 ± 4.4 years	Korean women	BPA concentration was significantly elevated in the DOR group.
Czubacka et al., 2021 [20]	Cross-sectional study	Fertility clinic	Urine samples	511 women	33.30 ± 3.69 years	Polish women	Significant association between BPA levels and diminished ovarian reserve.
Zhang et al., 2023 [21]	Cohort study	Shenyang, China	Urine samples	111 women	32.0 (interquartile range 4.1)	Chinese population	- BPA and BPS were significantly associated with an increased risk of diminished ovarian reserve. - BPS concentrations were negatively associated with AMH (β = 0.287, 95% CI: 0.505–0.070, *p* = 0.010).
Zhang et al., 2024 [22]	Cohort study	Shenyang, China	Urine samples	111 women	32.0 (interquartile range 4.1)	Chinese population	Higher urinary concentrations of BPA, BPF, and BPS were associated with lower AFC (β = 0.016; 95% CI: 0.025, 0.006 in BPA; β = 0.017; 95% CI: 0.029, 0.004 in BPF; β = 0.128; 95% CI: 0.197, 0.060 in BPS).
Zhan et al., 2023 [23]	Case-control study	Shandong, Shanghai, and Zhejiang Provinces, China	Urine samples	733 women	29 years	Chinese women	Exposure to BPA and its analog is correlated with a significantly higher risk of developing PCOS.
Patel et al., 2024 [24]	Case-control study	Ahmedabad, India	Blood samples in early follicular phase	130 women (80 women in the PCOS group and 50 controls)	29.16 ± 4.15 in the PCOS group and 24.34 ± 5.11 in Control group	Indian population	BPA levels of the PCOS group were significantly higher compared to the controls (102.15 ± 0.1 ng/mL vs. 61.35 ± 50.13 ng/mL, *p* < 0.0001).
Liang et al., 2020 [25]	Cross-sectional study	Center for Reproductive Medicine of Shandong University	Urine samples	111 women	28 years	Chinese women	Association between exposure to bisphenol A and S and oxidative stress as well as immune system imbalance in women with unexplained recurrent spontaneous abortion (URSA).
Studies investigating the effect of bisphenols on pregnancy outcomes							
Soomro et al., 2024 [26]	Cohort study	Calgary or Edmonton, Alberta, Canada	Urine samples	420 women	50.71% were in the 30 to 34 age range and 113 26.90% were over the age of 35	Canadian women	Significant association between BPA and the onset of gestational diabetes mellitus (GDM).
Tang et al., 2023 [27]	Case-control Study	Guangxi, China	Serum samples collected during the first trimester	500 women (100 women with GDM and 400 matched controls)	30.62 ± 6.46 for the cases and 30.6 ± 6.41 for controls	Chinese population	Bisphenol S exposure in first trimester is statistically significantly associated with an increased risk of developing gestational diabetes mellitus.
Zhu et al., 2022 [28]	Case-control Study	United States, Pregnancy Environment and Lifestyle Study (PETALS) cohort	Urine samples collected during the first and second trimester	333 women (111 women with GDM and 222 matched controls)	31.2 ± 4.6	Asian/Pacific Islander 39.6%, Black 9%, Hispanic 33.3%, White 14.1% and Other 3.9%	- GDM case subjects had a higher detection frequency of BPA in the second trimester (79.6 vs. 66.7%) and higher cumulative levels of BPS across the two trimesters (145.7 vs. 103.9 ng/mL × day). - First trimester urinary BPS was associated with higher risk of developing GDM (aOR: 2.12, 95% CI 1.00–4.50).
Trasande et al., 2024 [29]	Cohort study	United States, The NIH Environmental influences on Child Health Outcomes (ECHO) Program	Urine samples	3619 women	22.9% were less than 25 years old, 58.8% were between 25 and 34 years old, and 18.3% were more than 35 years old.	Non-Hispanic White (41.3%), Non-Hispanic Black (13.4%), Hispanic (34.5%) and Other (10.8%)	- Bisphenol S in third trimester was associated with Small for Gestational Age (OR 1.52, 95% CI 1.08, 2.13). - Bisphenol F in first and third trimester was significantly associated with Low Birth Weight.
Li et al., 2024 [30]	Cohort Study	Shenyang, China	Urine samples in the third trimester	113 women	56.6% were less than 30 years old and 43.4% were older than 30 years old	Han 84.1% and Other 15.9%	BPA and BPF exposure during pregnancy was significantly associated with lower birth weight (standardized regression coefficients (β = −0.081 kg, 95% CI: −0.134 to −0.027; β = −0.049 kg, 95% CI: −0.097 to −0.001)).
Hong et al., 2024 [31]	Cohort Study	Seoul, Republic of Korea	Maternal urine samples and cord blood samples	146 women	50.0% were less than 35 years old and 50.0% were older than 35 years old	Korean population	The asymmetric FGR group showed significantly higher maternal and fetal BPA levels compared to normal growth (*p* < 0.05 in both maternal urine and cord blood BPA).
Sol et al., 2021 [32]	Cohort Study	Rotterdam, The Netherlands	Maternal urine samples in all three trimesters	1379 women	30.5 (SD: 4.8)	European population	- First trimester bisphenol S was associated with larger fetal head circumference and fetal weight. - Bisphenol S and F in first trimester were also associated with a lower risk of being born at a small size for gestational age (OR = 0.56, 95% CI: 0.38–0.74 and OR = 0.55, 95% CI: 0.36–0.85, respectively).
Liang et al., 2021 [33]	Cohort Study	Guangxi, China	Maternal blood samples in first trimester	2023 women (113 in the preterm group and 1910 in term group)	28.2 ± 5.5 for the term group and 29.1 ± 5.9 for the preterm group	Chinese population	Bisphenol F (BPF) concentrations were positively associated with the risk of PTB (OR = 1.73, 95% CI: 1.18, 2.55).

BPA: Bisphenol A, BPF: Bisphenol F, BPS: Bisphenol S, DOR: diminished ovarian reserve, PCOS: polycystic ovarian syndrome, AMH: Anti-Müllerian Hormone, AFC: Antral Follicle Count, GDM: Gestational Diabetes Mellitus, PTB: Preterm Birth, FGR: Fetal Growth Restriction, SD: Standard Deviation, OR: Odds Ratio, aOR: adjusted Odds Ratio, CI: Confidence Interval.

## Data Availability

No new data were created or analyzed in this study.
